# LncRNA DICER1‐AS1 promotes colorectal cancer progression by activating the MAPK/ERK signaling pathway through sponging miR‐650

**DOI:** 10.1002/cam4.5550

**Published:** 2023-01-27

**Authors:** Wenfei Li, Chuanfeng Ke, Cuiyan Yang, Jieyao Li, Qikui Chen, Zhongsheng Xia, Jihao Xu

**Affiliations:** ^1^ Department of Gastroenterology Sun Yat‐Sen Memorial Hospital, Sun Yat‐Sen University Guangzhou Guangdong China; ^2^ Guangdong Provincial Key Laboratory of Malignant Tumor Epigenetics and Gene Regulation Sun Yat‐Sen Memorial Hospital, Sun Yat‐Sen University Guangzhou Guangdong China; ^3^ Department of Gastrointestinal Surgery The First Affiliated Hospital of Guangzhou Medical University Guangzhou China

**Keywords:** colorectal cancer, DICER1‐AS1, malignant proliferation, MAPK/ERK pathway, MAPK1, miR‐650

## Abstract

**Background:**

Colorectal cancer (CRC) is a disease with high morbidity and mortality rates globally. Long noncoding RNAs (lncRNAs) play a fundamental role in tumor progression, and increasing attention has been paid to their role in CRC. This study aimed to determine the function of lncRNA DICER1 antisense RNA 1 (DICER1‐AS1) in CRC and confirm its potential regulatory mechanisms in CRC.

**Methods:**

The publicly available dataset was used to assess DICER1‐AS1 function and expression in CRC. RT–qPCR or western blot assays were performed to verify DICER1‐AS1, miR‐650, and mitogen‐activated protein kinase 1 (MAPK1) expression in CRC cells or tissues. To determine the function of DICER1‐AS1, we performed CCK‐8, colony formation, transwell, cell cycle, and in vivo animal assays. Using RNA sequence analysis, luciferase reporter assays, and bioinformatics analysis, the connection between DICER1‐AS1, MAPK1, and miR‐650 was investigated.

**Results:**

DICER1‐AS1 was significantly upregulated in CRC tissue compared to normal colon tissue. High DICER1‐AS1 expression suggested a poor prognosis in CRC patients. Functionally, upregulation of DICER1‐AS1 effectively promoted CRC proliferation, migration, and invasion ex vivo and tumor progression in vivo. Mechanistically, DICER1‐AS1 functions as a competitive endogenous RNA (ceRNA) that sponges miR‐650 to upregulate MAPK1, promotes ERK1/2 phosphorylation, and sequentially activates the MAPK/ERK signaling pathway.

**Conclusion:**

Our investigations found that upregulation of DICER1‐AS1 activates the MAPK/ERK signaling pathway by sponging miR‐650 to promote CRC progression, revealing a possible clinically significant biomarker and therapeutic target.

## Graphical abstract

DICER1‐AS1 sponges miR‐650 to upregulate MAPK1 expression, promoting ERK1/2 phosphorylation and activating the MAPK/ERK pathway, thus promoting CRC progression.

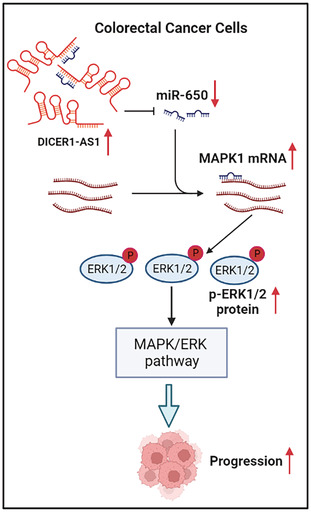

## 1 INTRODUCTION

Colorectal cancer (CRC) continues to be one of the most detrimental diseases affecting human health and is the third most frequently diagnosed and the second most lethal cancer worldwide.^1^, ^2^, ^3^ Clinically, CRC is usually diagnosed at an intermediate or late stage, and most patients have malignant hyperplasia and metastases. Moreover, despite advances in treatment strategies in recent years, including improved surgical techniques, chemotherapy, and targeted drugs, the prognosis of CRC patients is still not optimistic.^4^ Therefore, there is a need to further elucidate the mechanisms of colon carcinogenesis and progression to improve early diagnosis and find more treatment methods.

LncRNAs are noncoding RNAs that are more than 200 nucleotides in length and cannot encode proteins. There is growing evidence that lncRNAs can exert biological effects through a variety of mechanisms, such as serving as a guide for chromatin modification complexes, a scaffold for proteins, a decoy for mRNAs, and a sponge for miRNAs.^5^, ^6^ Especially, the role of lncRNAs in cancer progression through sponge for miRNAs has received increasing attention, for example, SNHG6 promotes colorectal cancer cell proliferation by sponging miR‐26a/b and miR‐214 to regulate EZH2 expression^7^; TINCR promotes hepatocellular carcinoma progression by sponging miR‐195‐3p to regulate ST6GAL1 expression^8^; and LINC00680 promotes esophageal squamous cell carcinoma progression by sponging miR‐423‐5p to regulate PAK6 expression.^9^ Moreover, there is strong evidence that abnormal expression or function of lncRNAs is strongly correlated with the development of human diseases, especially in cancer. A variety of biological functions can be performed by lncRNAs in human tumors, including cell proliferation, differentiation, migration, invasion, apoptosis, and resistance to drugs.^10^ For example, lncRNAs HOTAIR, GClnc1, SLCO1C1, FAM225A, and TINCR were proven to play a role in carcinogenesis,^8^, ^11^, ^12^, ^13^, ^14^ and in CRC, GLCC1 can stabilize c‐Myc to promote CRC progression.^15^ FEZF1‐AS1 promotes CRC tumor growth by activating the PKM2 signaling pathway.^16^ These studies indicate that lncRNAs play a significant role in human tumors by participating in the regulation of multiple intracellular processes.

DICER1‐AS1 was shown to be an oncogenic factor in osteosarcoma that promotes osteosarcoma cell progression by regulating the miR‐30b/ATG5 pathway.^17^ Hu et al. showed that DICER1‐AS1 is a suppressor molecule in pancreatic cancer that inhibits pancreatic cancer cell proliferation by promoting the maturation of miR‐5586‐5p.^18^ Moreover, Ma et al. elucidated that DICER1‐AS1 regulates the miR‐296‐5p/STAT3 pathway and thus promotes CRC progression ex vivo.^19^ However, DICER1‐AS1 in colon tumorigenesis remains unclear, and its role and mechanism in CRC require further study.

In our research, we demonstrated that DICER1‐AS1 is a cancer‐promoting factor that is significantly upregulated in CRC, and higher DICER1‐AS1 expression indicates a poor prognosis for CRC patients. Moreover, DICER1‐AS1 can promote CRC cell progression. As a ceRNA, DICER1‐AS1 binds to miR‐650, thereby increasing MAPK1 expression, promoting ERK1/2 phosphorylation and activating the MAPK/ERK signaling pathway. Our study suggests that DICER1‐AS1 is a clinically significant biomarker and therapeutic target.

## 2 MATERIALS AND METHODS

### 2.1 Clinical samples and cell culture

Our clinical samples were obtained from patients undergoing surgical resections at Sun Yat‐Sen Memorial Hospital between June and August 2020, and all of these recruited patients signed their written consent forms. Our study experimental protocols were all authorized by the Institutional Review Board Committee of Sun Yat‐Sen Memorial Hospital.

For cell culture, we purchased normal intestinal epithelial cells (NCM460) and human CRC cell lines (RKO, LOVO, HCT116, and SW480) from the American Type Culture Collection (ATCC). DMEM (Gibco) containing 10% fetal bovine serum (Gibco) was used for cell culture in an incubator at 37°C and 5% CO2.

### 2.2 RT–qPCR assays and separation of nuclear and cytoplasmic RNA

TRIzol reagent (Thermo Fisher Scientific), chloroform, isopropanol, and 75% ethanol were used to isolate total RNA from cell lines, and then A260/A280 values were measured to clarify the quality of the RNA. cDNA synthesis and quantitative PCR were performed as previously described.^20^ GAPDH or U6 was used as an internal reference gene, and the relative expression levels of the genes were calculated by the 2−△△ct method. All primer sequences were as follows (5′‐3′): GAPDH (ACAACTTTGGTATCGTGGAAGG, GCCATCACGCCACAGTTTC); DICER1‐AS1 (CATGTGTTGTGAGGGTTCTTCTG, TCCAGACCACACATCCATATTCC); MAPK1 (GCACCAACCATCGAGCAAAT, CTTGAGGTCACGGTGCAGAA). U6, hsa‐miR‐3612 and hsa‐miR‐650 primers were provided by RiboBio.

To isolate nuclear and cytoplasmic RNA, we first isolated total RNA with TRIzol reagent and then used nuclear and cytoplasmic protein extraction kits (KeyGEN BioTECH). DICER1‐AS1 expression was measured by RT–qPCR.

### 2.3 Cell transfection

We purchased lentivirus overexpressing DICER1‐AS1 from Hanbio Biotechnology (China). CRC cells were transfected with lentivirus overexpressing DICER1‐AS1 (DICER1‐AS1‐OE) and 5 mg/mL polybrene for 48 h. After transfection, the successfully transfected lentiviral vector cells were screened with 5 μg/mL puromycin (Cayman Chemical Company, USA). Then, we separated the single clone cells by puromycin and clarified the expression of DICER1‐AS1 by RT–qPCR assay. The clone with the highest DICER1‐AS1 expression was selected for subsequent experiments. The mimics of miR‐650 and negative controls were designed and supplied by RiboBio. We routinely performed cell transfection with miR‐650 and negative controls using Lipofectamine 3000 reagent (Invitrogen).

### 2.4 RNA sequencing

We extracted total RNA from SW480 cells transfected with lentivirus overexpressing DICER1‐AS1, and RNA sequencing was performed by Novogene (China). Differentially expressed genes were identified and displayed using scatter plots. The Gene Ontology (GO) database was used to describe the molecular functions, cellular environment, and biological processes involved in the differentially expressed genes. Pathway enrichment analysis of differentially expressed genes was performed using the Kyoto Encyclopedia of Genes and Genomes (KEGG) database.

### 2.5 Cell proliferation assay

For CCK‐8 assays, transfected cells (1 × 10^3^) were placed into 96‐well dishes for 4 days. Ten microliters of CCK‐8 reagent (APExBIO, USA) was added to each well and incubated for 2 h at 37°C, followed by absorbance readings at 450 nm every day. For the colony formation assay, transfected cells (2 × 10^3^) were inoculated into 6‐well plates, and the medium was changed every 3–5 days. After 14 days of incubation, each chamber was fixed, stained, and then photographed to determine the number of colony‐forming units.

### 2.6 Transwell assay

For migration assays, a membrane pore size of 8 mm (Corning, USA) transwell chambers were coated without Matrigel (Corning). Briefly, we added 200 μl of serum‐free DMEM suspension containing 2 × 10^5^ cells to the upper chamber, and 600 μl of 20% FBS media was placed into a 24‐well culture plate for incubation. After 48 h, the cells were fixed and stained, inverted microscope images were taken, and the average values were taken for statistical analysis. Matrigel was plated on top of the upper membrane for cell invasion, and the other steps were the same as those in the migration assays.

### 2.7 Western blot assay

Cells were harvested, lysed with RIPA lysis buffer (Beyotime, China), incubated, and centrifuged, and protein concentrations were assayed with a BCA assay kit (Beyotime, China). The expression levels of the proteins of interest were detected by western blotting as described previously.^21^ The primary and secondary antibodies were as follows: GAPDH rabbit mAb (1:3000, CST), p‐ERK1/2 rabbit mAb (1:2000, CST), and HRP‐conjugated anti‐rabbit IgG (1:1000, EpiZyme Biotechnology). These bands were detected with the Omni‐ECL kit (EpiZyme Biotechnology) in a MiniChemi 610 imaging system, and related data were analyzed by ImageJ.

### 2.8 Flow cytometry assay

Flow cytometry was used to analyze whether DICER1‐AS1 regulates the CRC cell cycle. The experimental protocol was as described previously.^22^ The cell phase distribution was measured using MODFIT LT3.1 software (Verity Software House, USA).

### 2.9 Fluorescence in situ hybridization (FISH)

A fluorescence in situ hybridization kit (RiboBio, China) was used to observe the location of DICER1‐AS1 in CRC cells. Briefly, the transfected cells were fixed at a density of approximately 60%–70%, permeated with Triton X‐100, blocked with prehybridization solution and incubated overnight at 37°C with Cy3‐labeled DICER1‐AS1 or 18 s FISH Probe Mix in darkness. Finally, DAPI (diamidino‐2‐phenylindole) was used to counterstain the nuclei.

### 2.10 Luciferase reporter assays

We predicted the 3′UTR of DICER1‐AS1 and MAPK1 with potential miR‐650 binding sites and their mutant forms from public databases and designed, synthesized, and cloned them into the psiCHECK2 dual‐luciferase vector from IGEbio (China), named DICER1‐AS1 (WT), DICER1‐AS1 (MUT), MAPK1 (WT), and MAPK1 (MUT), respectively. We cotransfected dual‐luciferase reporter plasmids with miR‐650 mimics or mimics control into SW480 and LOVO cells. After 48 h, a Duo‐LiteTM Luciferase Assay System (Vazyme, China) was used to detect the relative luciferase activities.

### 2.11 Animal experiments

For in vivo experiments, HCT116 cells (5 × 10^6^/0.1 ml PBS) transfected with lentivirus overexpressing DICER1‐AS1 and the negative control were inoculated subcutaneously into the right axilla and posterior flank of the same BALB/C nude mice (4–5 weeks old, male). Each tumor was measured every 6 days, and volume = length × width^2^/2 was applied to calculate tumor volume. The mice were euthanized 30 days later, and the subcutaneous tumors were harvested, weighed, and photographed. The experimental animals were approved by the Institutional Animal Care and Use Committee of Sun Yat‐Sen University.

### 2.12 Statistical analysis

GraphPad Prism 8 (GraphPad software package, USA) was applied for statistical analysis of the data and is shown as the mean ± standard deviation (SD) in this study. Student's *t*‐test or one‐way ANOVA was used to compare data between groups, and statistical significance was defined as *p <* 0.05.

## 3 RESULTS

### 3.1 DICER1‐AS1 is upregulated in CRC and correlates with poor patient prognosis

**FIGURE 1:**
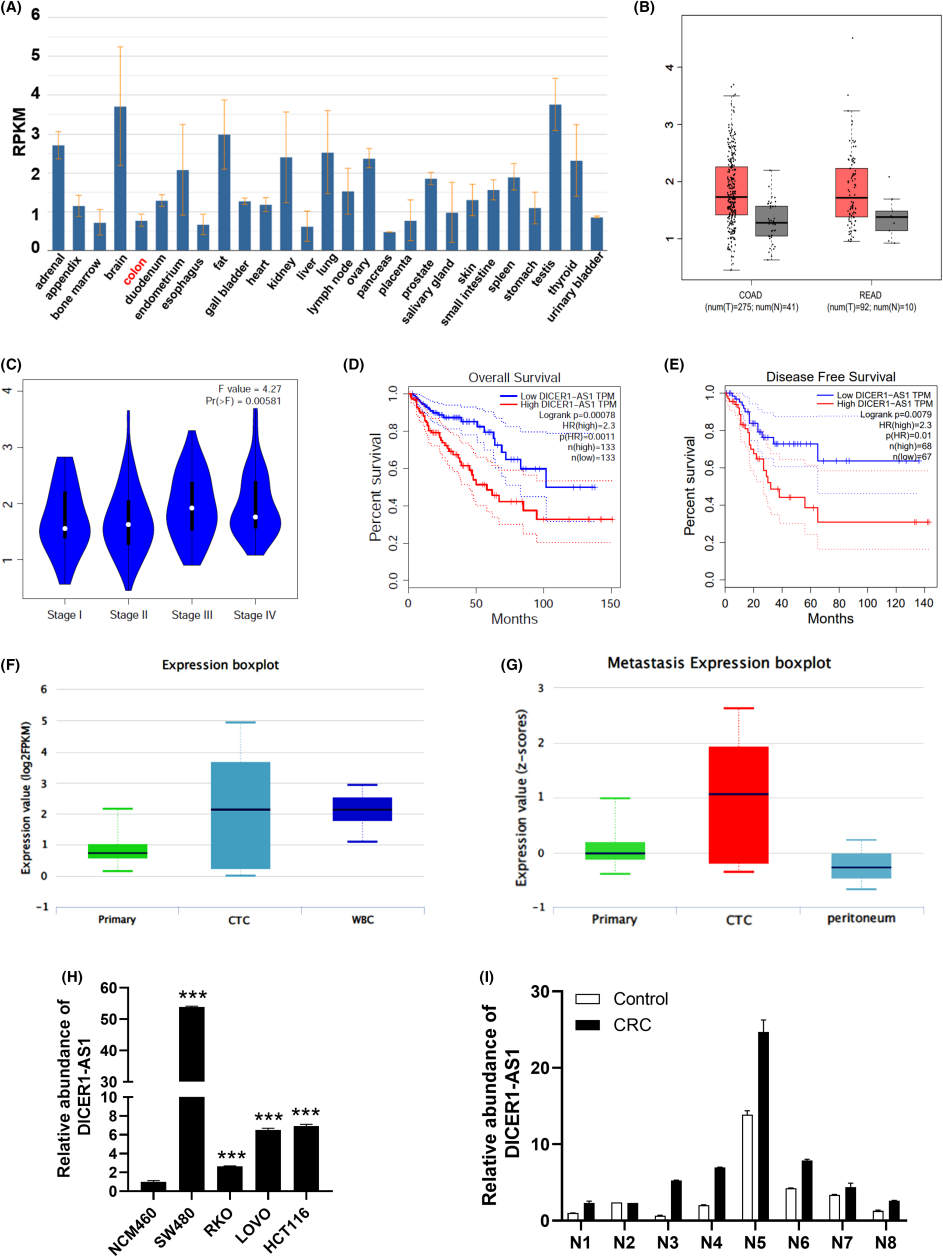
DICER1‐AS1 is upregulated in CRC and correlates with poor patient prognosis. (A) NCBI results of downregulated DICER1‐AS1 in normal colon tissues. (B) DICER1‐AS1 expression in human CRC and normal tissues was analyzed from TCGA database. (C) DICER1‐AS1 expression in all stages of CRC (*p* < 0.05). (D, E) Kaplan–Meier analysis of overall survival (D) and disease‐free survival (E) in two groups stratified by low and high expression of DICER1‐AS1 in CRC patients based on clinical data derived from the GEPIA database. (F‐G) DICER1‐AS1 overexpression is found in circulating cancer cells (CTCs) from CRC. (H) DICER1‐AS1 expression in CRC cell lines (SW480, RKO, LOVO, and HCT116) compared with the normal colorectal epithelial cell line NCM460 measured by RT–qPCR. (I) RT–qPCR analysis of DICER1‐AS1 expression in eight pairs of CRC and corresponding normal tissues. The data are presented as the mean ± SD, *p < 0.05, **p < 0.01, ***p < 0.001.

To discover the clinical significance of DICER1‐AS1, we analyzed its expression levels in publicly available databases. As shown in NCBI (https://www.ncbi.nlm.nih.gov/), DICER1‐AS1 was significantly downregulated in normal colon tissue (Figure 1A). The TCGA database revealed significantly higher expression of DICER1‐AS1 in COAD and READ tumor tissues than in normal tissues (Figure 1B). We further reviewed publicly available databases to explore the clinical significance of DICER1‐AS1 in CRC tumorigenesis. As shown in the TCGA database, patients in TNM stages III‐IV expressed more DICER1‐AS1 than those in TNM stages I‐II, suggesting that higher expression of DICER1‐AS1 was associated with more aggressive tumors (Figure 1C). The Kaplan–Meier survival analysis in the GEPIA database showed that higher DICER1‐AS1 expression was associated with lower overall survival (OS) and disease‐free survival (DFS) rates. (Figure 1D‐E). In addition, DICER1‐AS1 was overexpressed in circulating cancer cells (CTCs) from patients with CRC (Figure 1F‐G), suggesting the possibility that tumor metastasis could be enhanced by DICER1‐AS1. Next, RT–qPCR experiments were conducted on NCM460 and four cancer cell lines (SW480, RKO, LOVO, and HCT116) to detect the expression of DICER1‐AS1. Compared to normal colorectal epithelial cells, NCM460, LOVO, RKO, SW480, and HCT116 cells showed significantly higher DICER1‐AS1 expression levels (Figure 1H). To determine whether the clinical samples showed consistent trends, we performed RT–qPCR assays for eight pairs of CRC tissues and adjacent normal tissues, which revealed that DICER1‐AS1 levels were significantly higher in CRC tissues (Figure 1I). In conclusion, these findings indicate that DICER1‐AS1 may be involved in the pathogenesis and progression of CRC.

### 3.2 DICER1‐AS1 promotes CRC cell progression in vitro

**FIGURE 2:**
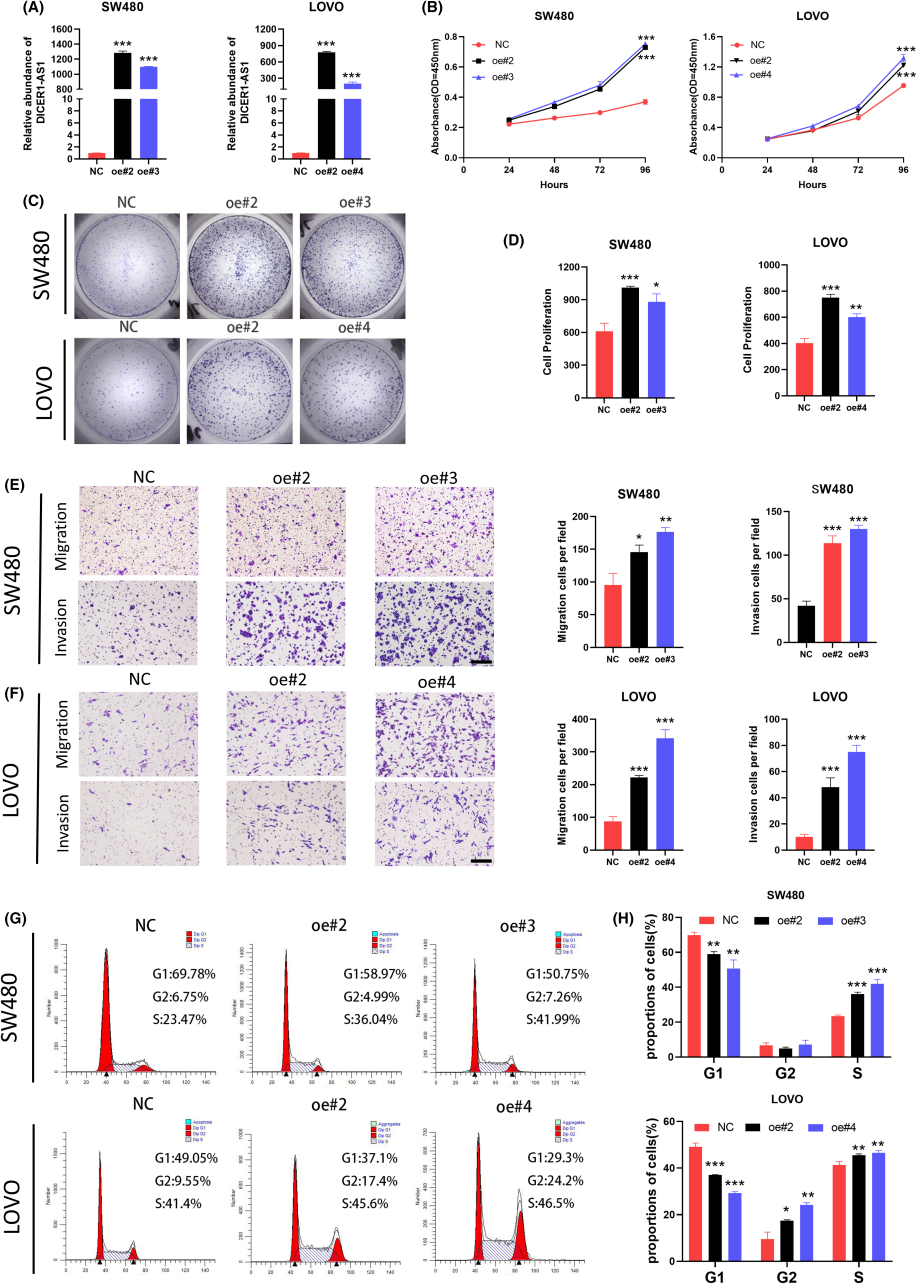
DICER1‐AS1 promotes CRC cell progression in vitro. (A) The relative DICER1‐AS1 expression level in SW480 and LOVO cells transfected with DICER1‐AS1‐OE or the negative control (NC). (B) CCK‐8 assay of SW480 and LOVO cells transfected with DICER1‐AS1‐OE or the negative control (NC). (C‐D) Representative images (C) and quantification (D) of the colony formation assay in SW480 and LOVO cells transfected with DICER1‐AS1‐OE or the negative control (NC). (E‐F) Representative images (left) and quantification (right) of transwell migration and invasion assays in SW480 and LOVO cells transfected with DICER1‐AS1‐OE or the negative control (NC) (magnification, ×100, scale bar, 500 μm). (G‐H) Representative images (G) and quantification (H) of flow cytometric analysis of the cell cycle in SW480 and LOVO cells transfected with DICER1‐AS1‐OE or the negative control (NC).The data are presented as the mean ± SD, *p < 0.05, **p < 0.01, ***p < 0.001.

To assess the biological effect of DICER1‐AS1 in CRC, lentivirus was stably transfected into SW480 and LOVO cells to construct a cell model of DICER1‐AS1 overexpression. We selected four lines of DICER1‐AS1‐OE cells, SW480 oe#2, SW480 oe#3, LOVO oe#2, and LOVO oe#4, for further study. Transfection efficiency was verified using RT–qPCR (Figure 2A). Then, the CCK‐8 assay demonstrated that upregulation of DICER1‐AS1 significantly promoted SW480 and LOVO cell proliferation (Figure 2B). Similarly, SW480 and LOVO cells were also found to have a marked increase in colony formation when DICER1‐AS1 was overexpressed (Figure 2C‐D). In addition, we explored whether DICER1‐AS1 promotes metastasis in CRC cells. As shown in the migration assay, upregulation of DICER1‐AS1 remarkably promoted the migration of SW480 and LOVO cells. In the invasion assay, the number of invaded cells was significantly increased in the DICER1‐AS1‐upregulated cells (Figure 2E, F). Furthermore, cell cycle analysis revealed an increase in the S phase fraction and a decrease in the G1 phase fraction with overexpression of DICER1‐AS1 (Figure 2G, H), suggesting that high DICER1‐AS1 can promote CRC cell cycle progression, which may be partially responsible for promoting cell proliferation. Taken together, DICER1‐AS1, as a carcinogenic factor, promotes CRC cell progression in vitro.

### 3.3 DICER1‐AS1 activates the MAPK/ERK signaling pathway

**FIGURE 3:**
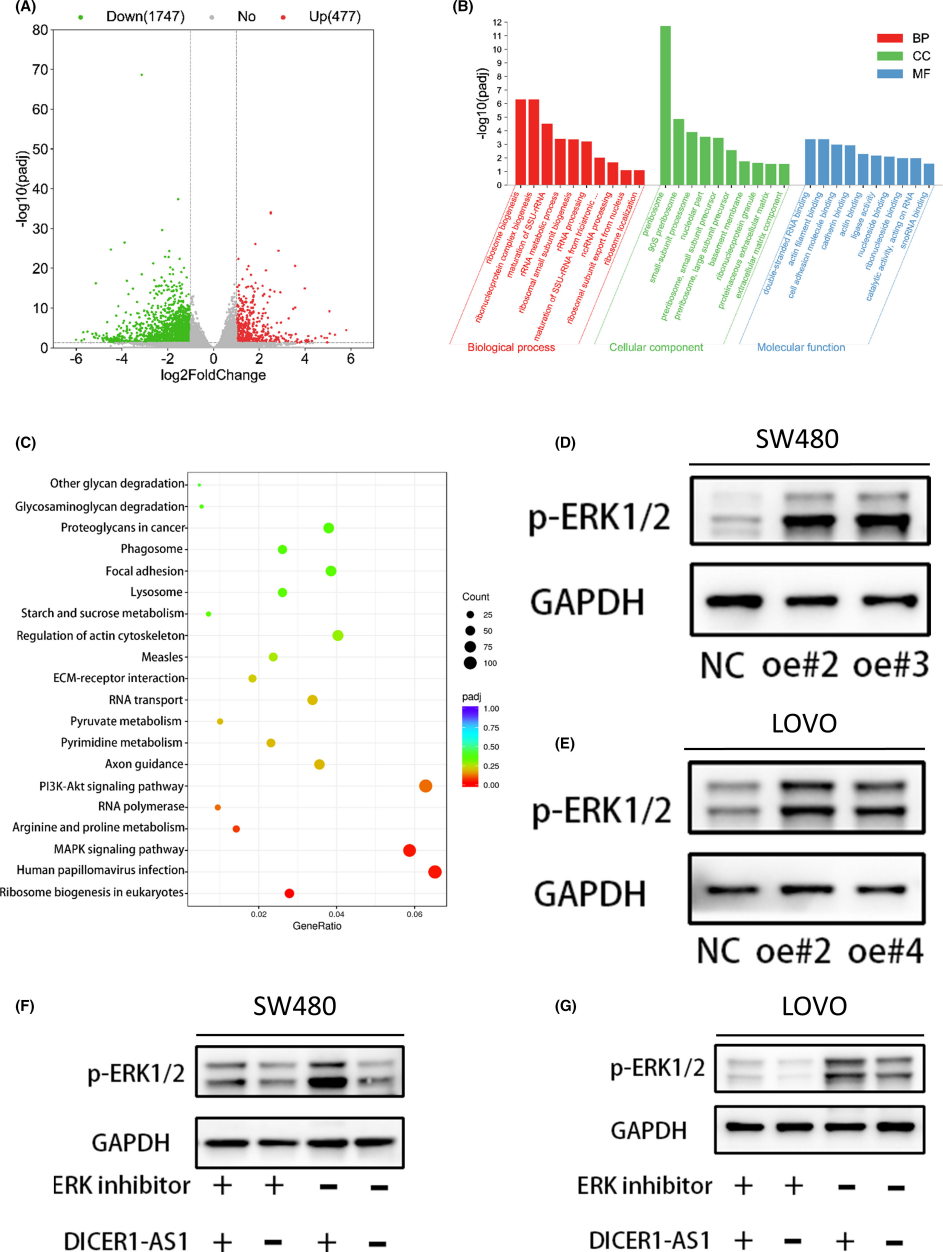
DICER1‐AS1 activates the MAPK/ERK signaling pathway. (A) Gene expression profiles of SW480 cells transfected with DICER1‐AS1‐OE or the negative control (NC). (B) GO functional annotation clustering of genes regulated by DICER1‐AS1 in SW480 cells is shown. The ten most enriched groups according to GO analysis are ranked based on *p* values. (C) Enriched KEGG pathway analysis showing the most enriched pathways. (D, E) Western blot analysis of p‐ERK1/2 expression in SW480 (D) and LOVO (E) cells transfected with DICER1‐AS1‐OE or the negative control (NC). (F, G) Western blot analysis of p‐ERK1/2 expression in SW480 (F) and LOVO (G) cells cotransfected with DICER1‐AS1‐OE or the negative control (NC) together with ERK inhibitor (80 nM). The data are presented as the mean ± SD, *p < 0.05, **p < 0.01, ***p < 0.001.

Having clarified that DICER1‐AS1 could promote CRC cell progression, we further explored its potential mechanisms. Therefore, we performed RNA sequencing analysis on DICER1‐AS1‐upregulated SW480 cells. In total, 477 genes were upregulated and 1747 genes were downregulated in the DICER1‐AS1‐upregulated SW480 group (∣FC∣ > 1 and P < 0.05) (Figure 3A). Interestingly, by analyzing the differentially expressed genes, we found that biological processes related to ncRNA processing were enriched, indicating that these genes may be associated with underlying mechanisms (Figure 3B). Furthermore, we performed KEGG pathway analysis of genes that are dysregulated due to DICER1‐AS1 upregulation. The results showed that the MAPK signaling pathway was significantly enriched (Figure 3C), which has been linked to increased tumorigenesis and progression in CRC.^23^, ^24^ Thus, we considered that DICER1‐AS1 may regulate CRC cells by activating the MAPK signaling pathway. Subsequently, phosphorylated ERK1/2 protein levels were markedly increased in DICER1‐AS1‐upregulated SW480 and LOVO cells, as shown by western blotting (Figure 3D, E), and ERK inhibitors partially alleviated these effects (Figure 3F, G). These data suggest that DICER1‐AS1 activates the MAPK/ERK pathway in CRC cells to promote tumorigenesis.

### 3.4 DICER1‐AS1 is predominantly distributed in the cytoplasm and competitively binds miR‐650

**FIGURE 4:**
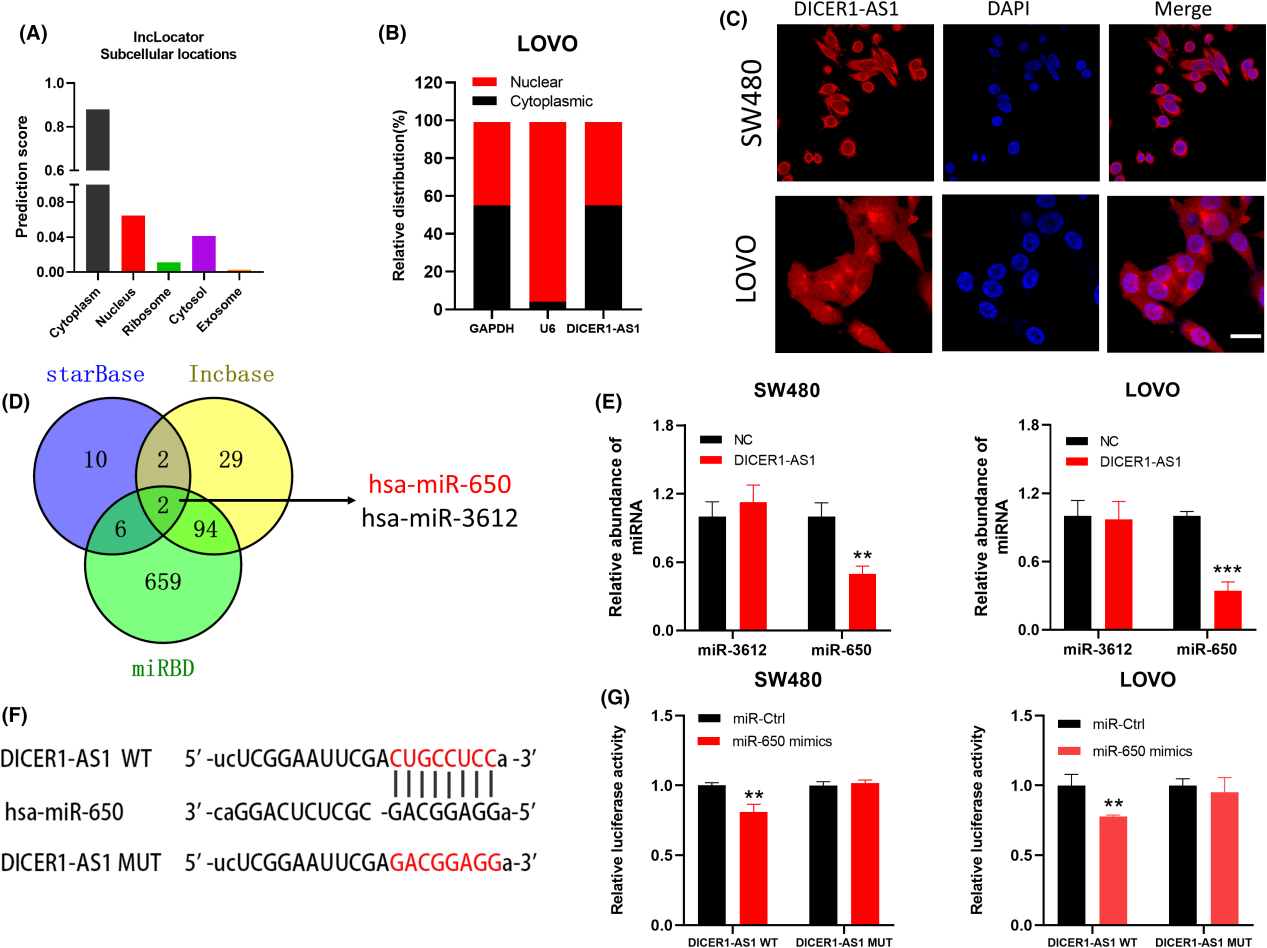
DICER1‐AS1 is predominantly distributed in the cytoplasm and competitively binds miR‐650. (A) DICER1‐AS1 was predicted to be located mainly in the cytoplasm using the bioinformatics tools in lncLocator. (B) A nuclear‐cytoplasmic fractionation assay indicated that DICER1‐AS1 was mainly localized in the cytoplasm of LOVO cells. (C) The localization of DICER1‐AS1 was observed in SW480 and LOVO cells by FISH. The nuclei were stained with DAPI (magnification, ×400, scale bar, 20 μm). (D) Two miRNAs were predicted to harbor complementary sequences to DICER1‐AS1 according to the lncbase, starBase and miRDB databases. (E) Relative levels of miR‐3612 and miR‐650 in SW480 and LOVO cells transfected with DICER1‐AS1‐OE or the negative control (NC). (F) Schematic diagram showing the sequence of miR‐650 and the 3′‐UTR of DICER1‐AS1, containing the wild type and mutant types. (G) Luciferase reporter assay indicated that DICER1‐AS1 WT activity was inhibited following transfection of SW480 and LOVO cells with miR 650 mimics. The data are presented as the mean ± SD, *p < 0.05, **p < 0.01, ***p < 0.001.

The specific mechanism by which DICER1‐AS1 activates the MAPK/ERK signaling pathway remains unclear. We predicted its subcellular localization in LncLocator (http://www.csbio.sjtu.edu.cn/cgi‐bin/lncLocator.py), which showed that DICER1‐AS1 was mainly distributed within the cytoplasm (Figure 4A). Then, we performed RT–qPCR of DICER1‐AS1 in both the nucleus and cytoplasm and a FISH assay to confirm its location (Figure 4B, C). Based on the distribution of DICER1‐AS1 in CRC cells, we hypothesized that DICER1‐AS1 functions as a ceRNA to sponge miRNA. We used the starBase, lncbase, and miRDB databases to identify its binding miRNAs. Two miRNAs (hsa‐miR‐3612 and hsa‐miR‐650) had potential complementary binding sequences with DICER1‐AS1 (Figure 4D). Then, we analyzed the relationship between miR‐3612, miR‐650 and DICER1‐AS1 expression in CRC cells by RT–qPCR, which showed that miR‐650 expression was decreased in CRC cells overexpressing DICER1‐AS1, but miR‐3612 expression was not affected (Figure 4E). Therefore, we considered miR‐650 to be a possible downstream gene of DICER1‐AS1. We predicted the possible binding sites between miR‐650 and the 3’‐UTR of DICER1‐AS1 using public databases and constructed the corresponding luciferase reporter vector, including DICER1‐AS1 wild type and mutant type (Figure 4F). We performed a dual‐luciferase reporter assay and revealed that upregulation of miR‐650 significantly reduced the luciferase activity of the wild‐type DICER1‐AS1 gene fragment compared to that of the mutant DICER1‐AS1 vector (Figure 4G). Based on the above results, we tentatively speculated that DICER1‐AS1 may exert oncogenic effects through competitive binding of miR‐650.

### 3.5 MiR‐650 inhibits CRC cell proliferation

**FIGURE 5:**
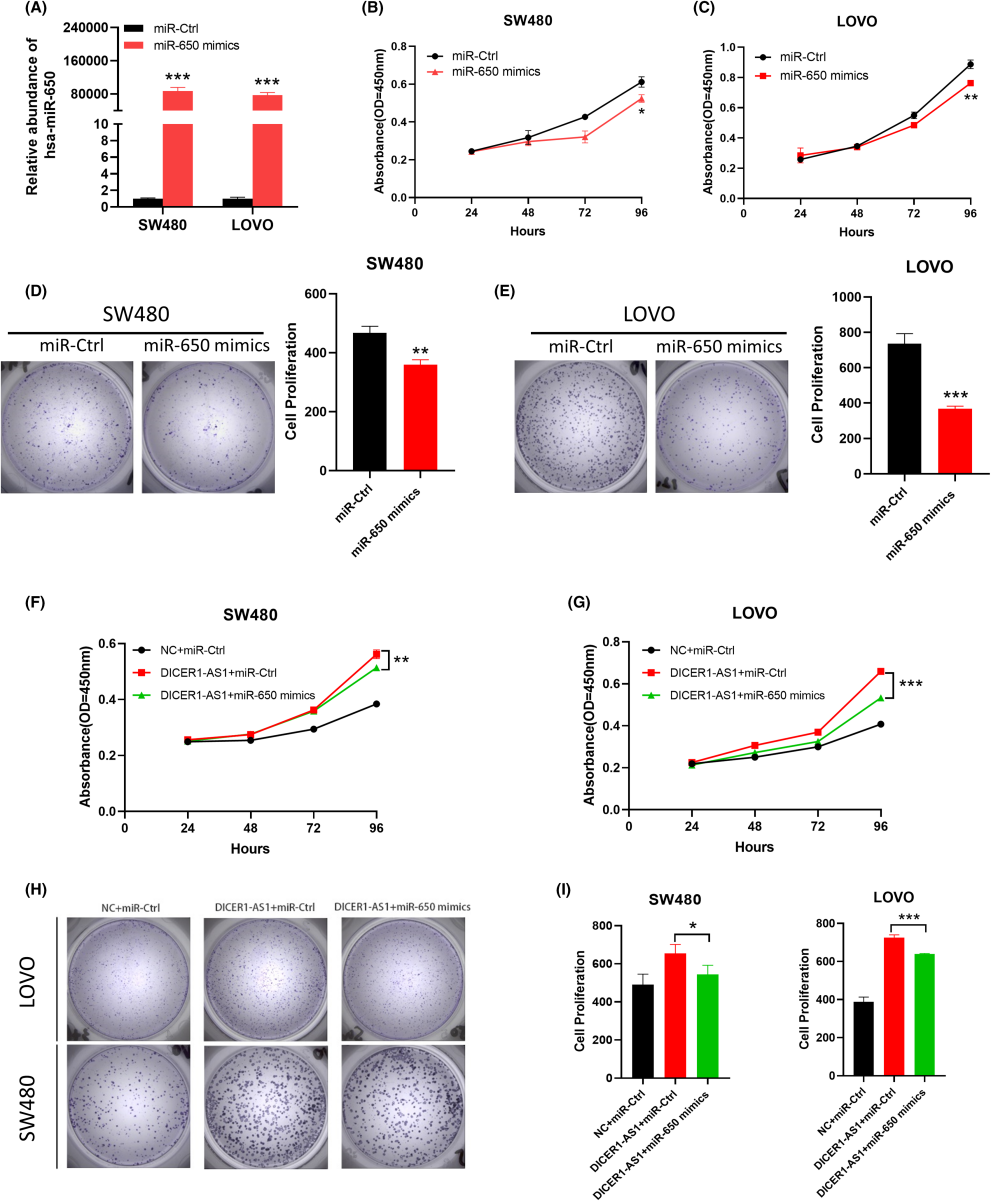
MiR‐650 inhibits CRC cell proliferation. (A) Expression level of miR‐650 in SW480 and LOVO cells transfected with miR‐650 mimics or miR‐Ctrl. (BC) CCK‐8 assay of SW480 and LOVO cells transfected with miR‐650 mimics or miR‐Ctrl. (D, E) Representative images (left) and quantification (right) of the colony formation assay in SW480 and LOVO cells with miR‐650 mimics or miR‐Ctrl. (F, G) CCK‐8 assays of SW480 (F) and LOVO (G) cells cotransfected with DICER1‐AS1‐OE or negative control (NC) together with miR‐650 mimics or miR‐Ctrl. (H, I) Representative images (H) and quantification (I) of the colony formation assay in SW480 and LOVO cells cotransfected with DICER1‐AS1‐OE or negative control (NC) together with miR‐650 mimics or miR‐Ctrl. The data are presented as the mean ± SD, *p < 0.05, **p < 0.01, ***p < 0.001.

We examined the function of miR‐650 in CRC cells by transfecting miR‐650 mimics and mimic controls into SW480 and LOVO cells to construct a cell model in which miR‐650 is overexpressed, and we used RT–qPCR to detect transfection efficiency (Figure 5A). By using the CCK‐8 assay and colony formation assay, we found that miR‐650 upregulation inhibited the growth of SW480 and LOVO cells (Figure 5B‐E). Furthermore, to confirm whether the function of DICER1‐AS1 was mediated by miR‐650, we transfected miR‐650 mimics and mimic controls into DICER1‐AS1‐overexpressing SW480 and LOVO cells. As shown in Figure 5F‐I, the proliferation ability of DICER1‐AS1‐overexpressing CRC cells was significantly affected by miR‐650 mimics. These results demonstrated that DICER1‐AS1 exerts a carcinogenic effect by competitively sponging miR‐650.

### 3.6 DICER1‐AS1 sponges miR‐650 to upregulate MAPK1 expression, promoting ERK1/2 phosphorylation, and activating the MAPK/ERK pathway

**FIGURE 6:**
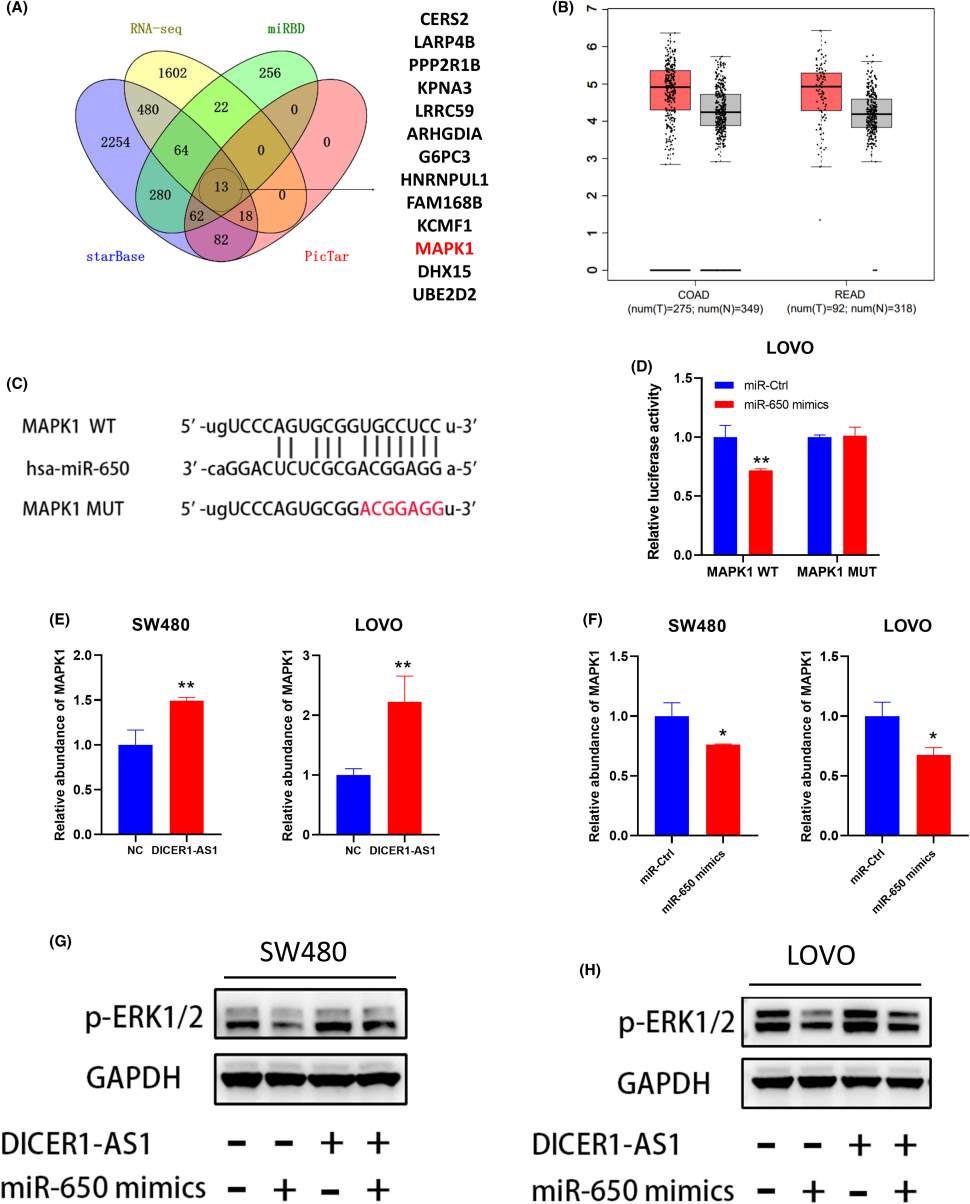
DICER1‐AS1 sponges miR‐650 to upregulate MAPK1 expression, promoting ERK1/2 phosphorylation and activating the MAPK/ERK pathway. (A) Venn diagram exhibiting the overlapping target genes of miR‐650 predicted by starBase, miRDB and PicTar and the uptrend of mRNA in RNA sequencing of SW480 cells transfected with DICER1‐AS1‐OE compared with the negative control (NC). (B) The GEPIA database showed an upregulated expression level of MAPK1 in CRC. (C) Schematic diagram showing the sequence of miR‐650 and the 3′‐UTR of MAPK1, containing the wild type and mutant types. (D) Luciferase reporter assay indicated that MAPK1 WT activity was inhibited following transfection of the cells with miR 650 mimics in LOVO cells. (E) Relative levels of MAPK1 in SW480 and LOVO cells transfected with DICER1‐AS1‐OE or the negative control (NC). (G) Relative levels of MAPK1 in SW480 and LOVO cells transfected with miR‐650 mimics or miR‐Ctrl. (H, I) Western blot analysis of p‐ERK1/2 expression in SW480 cells cotransfected with DICER1‐AS1‐OE or the negative control (NC) together with miR‐650 mimics or miR‐Ctrl. The data are presented as the mean ± SD, *p < 0.05, **p < 0.01, ***p < 0.001.

To identify the target genes of miR‐650 controlled by DICER1‐AS1, we performed bioinformatics prediction analysis in different databases, including PicTar, miRDB, and starBase. Figure 6A shows the overlapping genes, including miR‐650 target genes predicted by public databases and upregulated mRNAs from RNA sequencing of SW480 cells transfected with DICER1‐AS1‐OE. We found that MAPK1 was included, which is an important component of the MAPK/ERK signaling pathway. In addition, the GEPIA database showed an upregulated expression level of MAPK1 in CRC (Figure 6B). Furthermore, we predicted the possible binding region of miR‐650 to the 3’‐UTR of MAPK1 through public databases, constructed MAPK1 wild‐type and mutant luciferase reporter vectors, and performed dual‐luciferase reporter assays, which showed that miR‐650 mimics showed decreased luciferase activity of MAPK1 wild‐type gene fragments in LOVO cells compared with MAPK1 mutant vectors (Figure 6C, D). To determine whether DICER1‐AS1 activates the MAPK signaling pathway by relieving the repression of miR‐650 on MAPK1, we assessed the correlations between DICER1‐AS1, MAPK1, and miR‐650 expression levels in CRC cells by RT–qPCR. The results revealed that MAPK1 expression increased with overexpression of DICER1‐AS1 and decreased with overexpression of miR‐650 (Figure 6E, F). In addition, western blot analysis revealed that DICER1‐AS1 overexpression increased phosphorylated ERK1/2 protein levels, and this effect could be alleviated by the upregulation of miR‐650 (Figure 6G, H). Collectively, these results illustrated that DICER1‐AS1 sponges miR‐650 to upregulate MAPK1 expression, promoting ERK1/2 phosphorylation and activating the MAPK/ERK signaling pathway.

### 3.7 DICER1‐AS1 facilitates CRC cell tumorigenesis in vivo

To further clarify whether DICER1‐AS1 can facilitate CRC tumor growth in vivo, we performed in vivo nude mouse experiments. For the creation of a human CRC xenograft tumor model, equal amounts of HCT116 cells overexpressing DICER1‐AS1 and controls were inoculated subcutaneously into nude mice.

**FIGURE 7:**
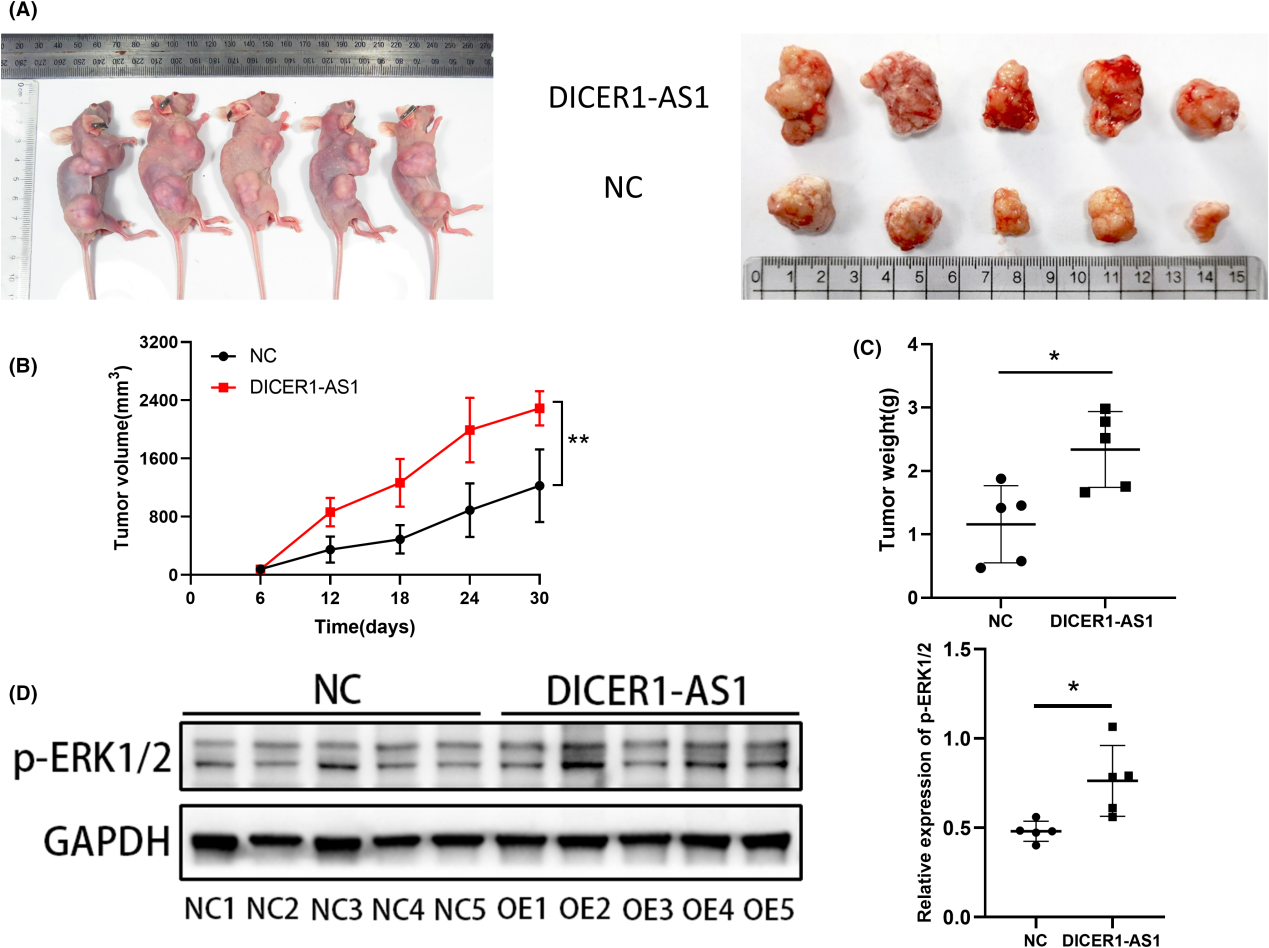
DICER1‐AS1 facilitates CRC cell tumorigenesis in vivo. (A) Imaging of mice (left) and xenograft tumors (right) showed that overexpression of DICER1‐AS1 led to larger tumors. (B, C) The nude mouse xenograft model showed that overexpression of DICER1‐AS1 promoted tumor growth (B) and tumor weights (C) compared with those of the negative control (NC) cells. (D) Relative expression levels of p‐ERK1/2 were observed in subcutaneous tumor tissues by western blot assays. The data are presented as the mean ± SD, *p < 0.05, **p < 0.01, ***p < 0.001.

There was a significant increase in weight and tumor size in the DICER1‐AS1 overexpression group compared with the control group (Figure 7A‐C). Moreover, we performed western blotting assay on subcutaneous tumor tissues and showed that there were higher levels of phosphorylated ERK1/2 proteins in the group overexpressing DICER1‐AS1, indicating that DICER1‐AS1 expression is positively correlated with MAPK/ERK activity (Figure 7D). In conclusion, these findings indicate that DICER1‐AS1 facilitates CRC cell tumorigenesis in vivo.

## 4 DISCUSSION

In the past decade, colorectal cancer (CRC) has remained one of the most common and lethal tumors, and it is still a worldwide problem affecting human health.^3^ Hence, a deep understanding of CRC development is essential. Recently, the role of long noncoding RNAs in human carcinomas, especially in CRC, has gained more attention. Our study revealed that lncRNA DICER1‐AS1 was aberrantly upregulated in CRC tissues and cell lines and that high levels were correlated with poor patient outcomes. DICER1‐AS1 functions as a ceRNA to bind miR‐650 to upregulate MAPK1 expression, promote phosphorylation of ERK1/2 and activate the MAPK/ERK pathway, thereby promoting CRC cell progression ex vivo and in vivo.

Several previous studies have reported that DICER1‐AS1 is associated with the tumorigenesis and progression of human tumors.^17^, ^18^, ^19^ However, there is a lack of clarity regarding the mechanism and role of DICER1‐AS1 in CRC. We first found the expression level and clinical significance of DICER1‐AS1 in CRC tumors and normal tissues by bioinformatics analysis, which showed that DICER1‐AS1 was expressed at low levels in normal colon tissues and significantly upregulated in CRC tissues; in addition, DICER1‐AS1 was strongly associated with disease progression and a poor prognosis. Then, we conducted functional experiments and showed that DICER1‐AS1 significantly promoted CRC progression both ex vivo and in vivo. Ma et al. also observed similar effects of DICER1‐AS1 on CRC cell lines.^19^ However, we validated that DICER1‐AS1 promoted CRC growth ex vivo, further indicating that it may be a therapeutic target in CRC.

Next, we performed RNA sequencing analysis on DICER1‐AS1‐upregulated SW480 cells to explore the mechanisms of DICER1‐AS1. An analysis of gene expression profiling revealed that overexpression of DICER1‐AS1 significantly activated MAPK signaling pathways, which are widely recognized as important to cell proliferation and migration of human cancers.^25^, ^26^ For example, exon 12‐containing LHX6 can facilitate cervical cancer cell growth by altering the MAPK signaling pathway^27^; SMIM30 promotes hepatocellular carcinoma development by MAPK pathway activation^28^; and BATF2 inhibits gastric cancer cell progression by inhibiting ERK signaling.^29^ In addition, mounting evidence has proven that activation of the MAPK pathway can promote CRC progression.^23^, ^30^, ^31^, ^32^ Therefore, we inferred that DICER1‐AS1 may promote CRC progression through the MAPK signaling pathway. Moreover, extracellular signal‐associated kinase (ERK1/2) is an important molecule that activates the MAPK/ERK pathway. We confirmed that high DICER1‐AS1 expression increased phosphorylated ERK1/2 protein levels by western blotting assays, and the addition of ERK inhibitors partially alleviated these effects, suggesting that DICER1‐AS1 activates the MAPK/ERK pathway to promote tumorigenesis in CRC.

To explore how DICER1‐AS1 activates the MAPK/ERK pathway, we investigated the localization of DICER1‐AS1 in CRC cells; it is mainly distributed in the cytoplasm according to RT–qPCR assays of cytoplasmic and nuclear RNA in CRC cells, FISH assays, and reliable public online databases. Thus, we considered that DICER1‐AS1 exerts its biological function through a ceRNA mechanism. LncRNAs can bind competitively to miRNAs, thereby indirectly regulating the expression of miRNA target genes.^33^ Interestingly, we proved that DICER1‐AS1 acts as a sponge for miR‐650 by bioinformatics prediction, RT–qPCR, and dual‐luciferase reporter assays. It has been previously shown that miR‐650 can function as a tumorigenic miRNA in various solid tumors. These include gastric cancer, oral cancer, prostate cancer, and anaplastic thyroid cancer.^34^ However, previous studies have reported that in CRC, miR‐650 not only inhibits tumor growth but also promotes tumor progression. For example, miR‐650 was downregulated in CRC as a tumor suppressor.^35^ A study from Zhou et al. showed that miR‐650 could directly target ANXA2, thereby promoting CRC cell proliferation.^36^ In our study, miR‐650 was shown to be a suppressor molecule of CRC and could inhibit cell growth, while we clarified the interrelationship between DICER1‐AS1 and miR‐650 in CRC by rescue experiments, which showed that miR‐650 inhibited the proliferation of CRC cells and partially rescued the overexpression of DICER1‐AS1.

Studies have shown that as ceRNAs, lncRNAs can exert biological functions by altering the target proteins of miRNAs. Our research reveals that MAPK1 is a downstream target of miR‐650 based on bioinformatics analysis, RNA sequencing data, and dual‐luciferase reporter experiments. In addition, miR‐650 was negatively correlated with MAPK1 expression at the mRNA level. MAPK1 is an important serine/threonine protein kinase in the MAPK/ERK pathway that activates the pathway by phosphorylating multiple substrates in the cellular compartment; consequently, cell proliferation, metabolism, and transcription are all affected by this process.^37^, ^38^ In recent research, MAPK1 has been identified as an important regulator of cancer‐related cell activities, including migration and proliferation, by activating MAPK signaling. For example, GK‐IT1 competitively binds to MAPK1 and activates the MAPK/ERK pathway by regulating MAPK1 phosphorylation, thereby promoting the progression of esophageal squamous cell carcinoma.^39^ LINC00483 promotes gastric cancer development by adsorbing miR‐490‐3p and upregulating MAPK1 expression.^40^ miR‐20a can inhibit breast cancer cell growth by directly targeting MAPK1 to inhibit the MAPK/ERK pathway.^41^ It has also been shown that miR‐422a inhibits cell growth by targeting MAPK1 in CRC.^42^ The present study demonstrated that MAPK1 was upregulated in CRC cells. Moreover, this was the first study to uncover the interaction of MAPK1 with miR‐650 and show that DICER1‐AS1 promotes CRC cell proliferation by competitively sponging miR‐650 to upregulate MAPK1 expression, promoting ERK1/2 phosphorylation, and activating the MAPK/ERK pathway.

## 5 CONCLUSION

To summarize, we identified DICER1‐AS1 as an upregulated lncRNA in CRC that is associated with a poor prognosis. Moreover, we found that DICER1‐AS1 could effectively promote CRC progression by acting as a ceRNA that sponges miR‐650 to upregulate MAPK1, promoting ERK1/2 phosphorylation, and activating the MAPK/ERK signaling pathway. Our findings demonstrate that DICER1‐AS1 may be a clinically significant biomarker and therapeutic target.

## AUTHOR CONTRIBUTIONS

**Wenfei Li:** Writing – original draft (lead); writing – review and editing (equal). **Chuanfeng Ke:** Data curation (equal). **Cuiyan Yang:** Resources (equal). **Jieyao Li:** Supervision (equal). **Qikui Chen:** Project administration (equal). **Zhongsheng Xia:** Project administration (equal). **Jihao Xu:** Writing – review and editing (equal).

## CONFLICT OF INTEREST

The authors declare that they have no conflict of interest.

## ETHICS STATEMENT

All the enrolled patients and their respective guardians had written consent prior to use the clinical samples and pathological features for research purposes. The overall protocol strictly adhered to guideline of the Institutional Review Board Committee at Sun Yat‐Sen Memorial Hospital, Sun Yat‐Sen University.

## CONSENT FOR PUBLICATION

All authors give consent for the publication of the manuscript in Cancer Medicine.

## Data Availability

Data sharing is not applicable to this article as no new data were created or analyzed in this study.
